# The role of PNPLA3 and TM6SF2 polymorphisms on liver fibrosis and metabolic abnormalities in Brazilian patients with chronic hepatitis C

**DOI:** 10.1186/s12876-021-01654-3

**Published:** 2021-02-23

**Authors:** Arthur Ivan N. Oliveira, Fernanda M. Malta, Patricia Momoyo Y. Zitelli, Ana Paula M. Salles, Michele S. Gomes-Gouvea, Ana Catharina S. Nastri, Joao Renato R. Pinho, Flair J. Carrilho, Claudia P. Oliveira, Maria Cássia Mendes-Corrêa, Mario G. Pessoa, Daniel F. Mazo

**Affiliations:** 1 Division of Clinical Gastroenterology and Hepatology, LIM07, Department of Gastroenterology, University of São Paulo School of Medicine (FMUSP), Av. Dr. Enéas de Carvalho Aguiar no 255, Instituto Central, # 9159, São Paulo, 05403-900 Brazil; 2 Department of Infectious Diseases, University of São Paulo School of Medicine (FMUSP), Av. Dr. Enéas de Carvalho Aguiar no 255, São Paulo, 05403-900 Brazil; 3 Division of Gastroenterology (Gastrocentro), School of Medical Sciences, University of Campinas (UNICAMP), Rua Carlos Chagas no 420, Campinas, 13083-878 Brazil

**Keywords:** Hepatitis C virus, Hepatitis virus, Genetic variation

## Abstract

**Background:**

Despite the growing body of knowledge about TM6SF2 and PNPLA3 polymorphisms in non-alcoholic fatty liver disease, their influence in the spectrum of HCV liver disease is not yet fully defined. Besides that, admixed populations, such as Brazilians, were not included in most of the studies.

**Methods:**

This cross-sectional study enrolled 365 treatment-naïve patients with HCV and 134 healthy individuals. TM6SF2 (rs58542926 c.499C > T) and PNPLA3 (rs738409 c.444C > G) polymorphisms were evaluated regarding their association with clinical and laboratory data, histological liver steatosis and fibrosis, and with components of the metabolic syndrome.

**Results:**

In HCV subjects, the frequencies of TM6SF2 CC and CT + TT were 89% and 11%, while PNPLA3 frequencies of CC and CG + GG were 51.4% and 48.6%. In the univariate logistic regression analysis, the TM6SF2 CT + TT genotype in HCV was associated with significant liver fibrosis (*p* = 0.047; OR 1.953; 95% CI 1.009–3.788). In comparison to the CT + TT genotype, the TM6SF2 CC genotype in HCV was associated with older age (*p* = 0.002), higher frequency of arterial hypertension (*p* = 0.032), obesity (*p* = 0.030), metabolic syndrome (*p* = 0.014) and lower total cholesterol levels (*p* = 0.036). The PNPLA3 GG subjects had lower body mass index than CG/ CC individuals (*p* = 0.047). None of the polymorphisms, or their combinations, was independently associated with hepatic steatosis or fibrosis. On the other hand, older age, lower serum levels of total cholesterol, and higher serum levels of alanine aminotransferase and alkaline phosphatase were associated with liver fibrosis in the multivariate logistic regression analysis.

**Conclusion:**

In this evaluation of an admixed HCV population, neither TM6SF2 nor PNPLA3 polymorphisms were independently associated with hepatic steatosis or fibrosis. Other factors seem more influential than these specific polymorphisms in isolation. More studies are warranted to clarify the role of the TM6SF2 and PNPLA3 polymorphisms in Brazilians with HCV.

## Background

Chronic hepatitis C virus (HCV) infection affects about 1% of the world population and remains one of the main causes of liver disease worldwide [1]. In Brazil, it is estimated that 0.53% of the total population is anti-HCV positive [2]. Between 55 and 85% of patients exposed to the virus develop chronic liver disease, and 15 to 20% of these will reach the stage of cirrhosis [3]. HCV is the leading cause of death among viral hepatitis in Brazil, and it remains a public health problem: in 2019, it was estimated that 22,747 individuals were infected with HCV [4].

Persistent HCV infection leads to the induction of pro-inflammatory cytokine production by Kupffer cells, activation of hepatic stellate cells, and development of liver fibrosis and cirrhosis [5]. Liver disease progression with accelerated fibrosis and cirrhosis is also associated with other factors, such as insulin resistance, obesity, non-alcoholic fatty liver disease (NAFLD), alcohol consumption, and coinfections with HIV or hepatitis B virus [6]. Indeed, metabolic syndrome (MetS) features, such as obesity and diabetes mellitus can negatively impact chronic HCV infection [7]. In addition, liver steatosis is another frequent complication in HCV-infected patients, affecting 50 to 72% of this population [8].

With the advent of genome-wide association studies (GWAS), the influence of genetic factors in the development of steatosis and progression of chronic liver disease has gained evidence, mainly in NAFLD [9–11]. In this context, the single nucleotide (SNP) polymorphisms E167K of the Transmembrane 6 Superfamily Member 2 (TM6SF2) gene, and the polymorphisms I148M of the Patatin-like Phospholipase-3 (PNPLA3) gene, are two of the most frequently studied polymorphisms across populations [9, 11].

Experimental and observational studies have shown that the E167K variant (T allele) is associated with TM6SF2 functional loss, with consequent decreased VLDL secretion and increased intrahepatic fat aggregation, conferring an increased risk for NAFLD [12–14]. The common allele (C allele), in turn, seems to be associated with a lower risk of steatosis, but with higher serum levels of total cholesterol, LDL, and triglycerides (TG), and increased risk of cardiovascular disease [15]. In patients with chronic hepatitis C, TM6SF2 is believed to regulate the interaction between viral glycoproteins and the host’s lipid metabolism, with potentially deleterious consequences for the liver [16]. Despite this theoretical association, studies on hepatitis C have shown inconsistent results [16–18].

The I148M variant of PNPLA3 has shown an association with the progression of liver fibrosis and steatosis, and in the occurrence of hepatocellular carcinoma [9, 19, 20]. The strongest associations with the severity of liver disease were seen in patients with NAFLD, including the Brazilians [21–23]. Studies on chronic hepatitis C corroborate the relationship of this polymorphism with liver disease progression and steatosis [19, 24, 25]. However, other studies with a predominance of Asian populations have shown divergent results [26, 27].

Despite the growing investigational experience with TM6SF2 and PNPLA3 polymorphisms in NAFLD, their role in the spectrum of liver disease by the hepatitis C virus is not yet fully defined. Also, there is little data in mixed populations, such as among Brazilians [28]. In this context, the aim of this study is to describe the prevalence of TM6SF2 and PNPLA3 polymorphisms in Brazilian patients with chronic hepatitis C naïve for antiviral therapy, and to evaluate their association with liver fibrosis, steatosis, and other components of the metabolic syndrome.

## Methods

### Clinical Design and Patients’ Selection

This was a single-center cross-sectional study conducted in healthy subjects and in patients with chronic hepatitis C, followed between 2014 and 2016 at the Outpatient Units of the Departments of Gastroenterology and Infectious Diseases at the Hospital das Clinicas of the University of Sao Paulo School of Medicine, Brazil. PNPLA3 (rs738409 c.444 C > G) and TM6SF2 (rs58542926 c.499C > T) polymorphisms were investigated in 365 patients with chronic hepatitis C naïve to antiviral therapy and in 134 unrelated healthy volunteers without chronic hepatitis B, C, or liver enzyme abnormalities.

The inclusion criteria for the HCV group were age ≥ 18 years old, presence of chronic hepatitis C, defined by HCV polymerase chain reaction (PCR) RNA positivity for at least 6 months, with histopathological diagnosis of chronic HCV, and naïve to antiviral therapy. All patients with chronic hepatitis C had liver histology evaluated by an experienced liver pathologist. The exclusion criteria were any other diagnosed chronic liver disease, significant alcohol intake (> 100 g/week), HIV or hepatitis B infection, previous liver transplantation, or refusal to participate in the study. The healthy volunteers’ group consisted of subjects without chronic hepatitis B, C, or liver enzyme abnormalities, selected from among the general population. This group was used only to describe the frequency of the PNPLA3 and TM6SF2 polymorphisms in healthy individuals in Brazil. No statistical analyses were performed between this group and patients with HCV.

### Variables evaluated

Demographic and anthropometric data (age, gender, self-reported ethnicity, and body mass index) were obtained from the medical charts, as well as the presence of comorbidities [arterial hypertension, diabetes mellitus, dyslipidemia, MetS, previous cardiovascular event (CVE), and hypotireoidism]. CVE was defined as any one of the following: previous acute myocardial infarction, stroke, or diagnosed peripheral arterial disease. Alcohol consumption was also registered.

Diagnosis of metabolic syndrome was based on the recommendations of the Adult Treatment Panel III Report. It was made when three or more of the following criteria were met: triglycerides ≥ 150 mg/dL, high-density lipoproteins (HDL) < 40 mg/dL (men) or < 50 mg/dL (women), fasting glucose ≥ 110 mg/dL, systolic blood pressure ≥ 130 mm Hg, and diastolic blood pressure ≥ 85 mm Hg and abdominal obesity [29]. The definition of insulin resistance was based on the calculation of the Homeostatic Model of Assessment (HOMA-IR), with insulin resistance defined by a HOMA-IR when ≥ 2.5 [30].

Serum biochemistry included: alanine aminotransferase (ALT), aspartate aminotransferase (AST), alkaline phosphatase, gamma-glutamyl transferase (GGT), creatinine, total cholesterol, high-density lipoprotein (HDL), low-density lipoprotein (LDL), and triglycerides. These were collected after a 12-h overnight fasting period at the time of the liver biopsy.

The serum HCV-RNA real-time PCR was made with the Amplicor HCV Monitor 2.0 test (Diagnostic Systems, Branchburg, NJ, USA, detection limit: 50 IU/ml). HCV genotyping was performed using the Versant^®^ HCV Genotype 2.0 (LiPA) test (Immunogenetics, Ghent, Belgium).

Liver histology was assessed by blinded experienced liver pathologists and considered the METAVIR classification for fibrosis and inflammation [31]. The evaluation of fibrosis (F) and inflammatory activity (A) was according to the following parameters: Fibrosis: F0 (absent), F1 (portal fibrosis without septa), F2 (portal fibrosis with rare septa), F3 (numerous septa without cirrhosis) and F4 (cirrhosis); inflammatory activity: A0 (absent), A1 (mild activity), A2 (moderate activity) and A3 (intense activity) [31]. Steatosis was classified as absent (affecting less than 5% of liver parenchyma) or present (more than 5%).

### DNA extraction and genotyping

Genomic DNA was isolated from 200 μl of blood and analyzed by QIAamp DNA Blood Mini Kit (QIAGEN, Hilden, Germany). Briefly, as previously described [22], PNPLA3 (rs738409 c.444 C > G) and TM6SF2 (rs58542926 c.499C > T) polymorphisms were genotyped using TaqMan primers and probes for allelic discrimination (7500 Fast Real-Time PCR system, Applied Biosystems, Thermo Fisher Brand, Foster City, California, USA) as per the manufacturer’s recommendations. Direct genotyping in random samples was conducted to validate the results. Quality control was conducted to check the reproducibility of the results.

### Ethical considerations

The Ethics Committee of the Hospital das Clinicas of the University of Sao Paulo School of Medicine has approved this study (numbered 1,531,382 and 2,676,105). The protocol followed the 1975 Declaration of Helsinki. Informed consent was obtained from participants.

### Statistical analysis

The data were compiled in a Windows Excel table for statistical analysis. Descriptive statistics (means, standard deviations) were calculated. The chi-square test and Fisher’s exact test were used to compare the categorical variables; Mann–Whitney and Kruskal–Wallis tests were used for numerical variables, since these did not have a normal distribution. The maximum percentage of missing values for any clinical/ biochemical covariate was 1.9%. Regarding liver histology covariates, we had one missing value for fibrosis, 11 for steatosis, and 65 for inflammation. To evaluate factors associated with liver fibrosis, inflammation, and steatosis, logistic regression analysis was used. In the multivariate logistic regression analysis, a stepwise variable selection criterion was used. Odds ratio (OR) and 95% confidence interval (CI) were calculated to evaluate the impact of these factors. Hardy–Weinberg equilibrium was evaluated. Heterozygosity and the polymorphic information content (PIC) in the studied groups were calculated [32]. The combination effect of TM6SF2 and PNPLA3 polymorphisms was evaluated on logistic regression analyses. A probability value of < 0.05 was considered significant. The Statistical Analysis System (SAS) for Windows software package, version 9.4 (SAS Institute Inc, 2002–2008, Cary, NC, USA) was used for the statistical analyses conducted by biomedical statisticians from the Statistics Service at School of Medical Sciences of the University of Campinas.

## Results

We included 365 patients with chronic hepatitis C and 134 healthy individuals in the study. Among the patients with HCV, 55.1% were women, the mean BMI was 27.57, and the mean age was 54.54 ± 12.7 years. The most frequent self-reported ethnicity was white (76.7%), and 23.18% of the subjects fulfilled the MetS criteria. Regarding HCV infection, the most prevalent genotype was 1 (79.4%), followed by genotype 3 (17.9%). All stages of liver fibrosis were represented in this cohort, with cirrhosis predominating (30.5%). The main characteristics of the included patients are detailed in Table 1.

**Table 1:** Demographic, clinical and biochemical characteristics of patients with HCV

	HCV patients (n = 365)
	% (n) or mean ± SD
Age (years)	54.54 ± 12.7
Men /Women (n)	44.9% (164) / 55.1% (201)
*Race (n)*	
White	76.7% (280)
Multiracial (Brown)	13.8% (51)
Black	9.3% (34)
Type 2 diabetes (n)	23.1% (83/359)
Dyslipidemia (n)	20.1% (72/358)
High-blood pressure (n)	43.5% (156/359)
Obesity (BMI ≥ 30)	29.4% (106/360)
BMI	27.57 ± 5.02
Metabolic Syndrome (n)	23.2% (83/358)
Hypothyroidism (n)	17.3% (62/359)
Previous alcohol use (n)	8.4% (30/359)
Previous CVE (n)	5.0% (17/359)
*HCV genotype (n)*	
1	79.4% (289/364)
2	2.2% (8/364)
3	17.9% (65/364)
4	0.5% (2/364)
HOMA-IR	3.18 ± 4.24
AST (U/L)	63.36 ± 50.84
ALT (U/L)	68.84 ± 51.27
Alkaline phosphatase (U/L)	83 ± 34.62
GGT (U/L)	102.07 ± 110.32
Creatinine (mg/dL)	0.89 ± 0.6
Total cholesterol (mg/dL)	157.25 ± 36.06
HDL (mg/dL)	52.11 ± 16.31
LDL (mg/dL)	85.01 ± 31.22
Triglycerides (mg/dL)	101.81 ± 52.5
*Liver histology*	
Fibrosis (n = 364)	
F0	7.4% (27)
F1	28.0% (102)
F2	18.1% (66)
F3	15.9% (58)
F4	30.5% (111)
* Inflammation (n = 300)*	
A0	5.7% (17)
A1	40.3% (121)
A2	36.7% (110)
A3	17.3% (52)
* Steatosis (n = 354)*	
0	44.9% (159)
1	32.5% (115)
2	16.7% (59)
3	5.9% (21)

ALT: alanine aminotransferase; AST: aspartate aminotransferase; BMI: body mass index; CVE: cardiovascular event; HDL: high-density lipoprotein; HOMA-IR: Homeostatic Model of Assessment; LDL: low-density lipoprotein; GGT: gamma glutamyl transferase; HCV: hepatitis C virus; SD: standard deviation

TM6SF2 and PNPLA3 polymorphisms were in Hardy–Weinberg equilibrium among all groups. In the HCV group, TM6SF2 and PNPLA3 heterozygosity were 0.20 and 0.56, and the PIC were 0.18 and 0.56, respectively. Among healthy subjects, the TM6SF2 and PNPLA3 heterozygosity were 0.13 and 0.58, and the PIC were 0.12 and 0.50, respectively. In healthy subjects, the frequencies of TM6SF2 CC and CT + TT were 93.3% and 6.7%, respectively. In patients with HCV, these frequencies were 89% and 11% (Table 2). Regarding PNPLA3 polymorphisms in healthy subjects, the frequencies of CC and CG + GG were 49.3% and 50.7%, respectively, and 51.4% and 48.6% in patients with HCV (Table 2). Analyzing the associations of PNPLA3 polymorphisms in patients with HCV, we found that GG subjects had lower BMI than CG/ CC individuals (25.19 ± 3.7 *vs.* 27.9 ± 5.24 *vs.* 27.67 ± 4.94, *p* = 0.046), and that the presence of MetS was more frequent in CC/CG subjects (*p* = 0.013) (Table 3). No differences were observed regarding any other clinical, biochemical, or liver histology variables related to PNPLA3 genotypes (Table 3), or with the presence/absence of the G allele (data not shown). On the other hand, when analyzing TM6SF2 polymorphisms in patients with HCV, the CC genotype was associated with older age (*p* = 0.002), higher frequency of arterial hypertension (*p* = 0.032), obesity (*p* = 0.030), MetS (*p* = 0.014), and higher serum levels of alkaline phosphatase (*p* = 0.041) and total cholesterol (*p* = 0.036), relative to the CT/TT genotype. Also, the presence of the TM6SF2 T allele was associated with significant liver fibrosis (≥ stage 2 fibrosis; *p* = 0.044) (Table 4).

**Table 2:** PNPLA3C > G and TM6SF2C > T frequencies in healthy subjects and in patients with HCV

	Genotype frequency % (n)			Total %
	CC	CG	GG	
PNPLA3				
Healthy subjects (n = 134)	49.3 (66)	41.0 (55)	9.7 (13)	100
HCV (n = 365)	51.4 (187)	41.2 (150)	7.4 (27)	100

TM6SF2	CC	CT	TT	
Healthy subjects (n = 134)	93.3 (125)	6.7 (9)	0	100
HCV (n = 365)	89.0 (322)	10.5 (38)	0.5 (2)	100

**Abbreviations:** HCV: hepatitis C virus

**Table 3:** Characteristics of patients with HCV according to PNPLA3 polymorphisms (n = 364)

	CC	CG % / (n) or mean ± SD	GG	*P* value
Age (years)	53.75 ± 12.9	55.94 ± 12.52	52.04 ± 12.03	0.191
Men /Women (n)	48.7% (91)/51.3% (96)	42% (63)/58% (87)	33.3% (9)/66.7% (18)	0.218
*Race*				
White	75.9% (142)	77.3% (116)	77.8% (21)	0.699
Multiracial (Brown)	15.5% (29)	13.3% (20)	7.4% (2)	
Black	8.6% (16)	9.3% (14)	14.8% (4)	
Type 2 diabetes (n)	23.4% (43)	25.7% (38)	7.7% (2)	0.133
Dyslipidemia (n)	21.2% (39)	21.1% (31)	3.9% (1)	0.103
High-blood pressure (n)	42.9% (79)	45.3% (67)	38.4% (10)	0.786
Obesity (BMI ≥ 30) (n)	30.8% (57)	30.4% (45)	15.4% (4)	0.259
BMI	27.67 ± 4.94	27.9 ± 5.24	25.19 ± 3.7	0.046* (GG < CG/CC)
Metabolic syndrome (n)	25.7% (47)	24.3% (36)	0% (0)	0.013*
Hypothyroidism (n)	17.4% (32)	16.9% (25)	19.2% (5)	0.958
Previous alcohol use (n)	8.7% (16)	7.4% (11)	7.7% (2)	0.913
HOMA-IR value ≥ 2.5 (n)	51.9% (97)	55.3% (83)	44.4% (12)	0.547
HOMA-IR	3.19 ± 4.65	3.33 ± 3.94	2.46 ± 2.71	0.293
AST (U/L)	66.11 ± 59.4	59.62 ± 37.57	66.63 ± 51.98	0.929
ALT (U/L)	71.82 ± 55.08	64.68 ± 43.82	73 ± 62.14	0.505
Alkaline phosphatase (U/L)	84.61 ± 33.1	82.71 ± 38.26	73.26 ± 20.73	0.196
GGT (U/L)	103.74 ± 115.94	104.09 ± 110.33	78.89 ± 62.5	0.761
Creatinine (mg/dL)	0.9 ± 0.77	0.87 ± 0.35	0.88 ± 0.39	0.956
Total cholesterol (mg/dL)	157.32 ± 37.85	158.26 ± 35.08	151.96 ± 29.2	0.752
HDL (mg/dL)	51.34 ± 16.01	52.32 ± 16.52	56.33 ± 17.43	0.466
LDL (mg/dL)	85.21 ± 32.12	85.89 ± 30.46	79.22 ± 30.06	0.541
Triglycerides (mg/dL)	104.16 ± 46.95	101.93 ± 60.93	86.7 ± 34.53	0.117
*Liver histology*				
Fibrosis (n) 0–1	35.3% (66)	35.3% (53)	37.0% (10)	
2–4	64.7% (121)	64.7% (97)	63.0% (17)	0.983
Inflammation (n) 0–1	44.2% (69)	46.6% (55)	52% (13)	
2–3	55.8% (87)	53.4% (63)	48% (12)	0.750
Steatosis (n) 0	48.7% (91)	41.2% (61)	33.3% (9)	
1–3	51.3% (96)	58.8% (87)	66.7% (18)	0.190

Chi-square test, Fisher's exact test, Mann–Whitney, and Kruskal–Wallis test

ALT: alanine aminotransferase; AST: aspartate aminotransferase; BMI: body mass index; HCV: hepatitis C virus; HDL: high-density lipoprotein; HOMA-IR: Homeostatic Model of Assessment; LDL: low-density lipoprotein; GGT: gamma glutamyl transferase; SD: standard deviation

*p value < 0.05

**Table 4:** Characteristics of patients with HCV according to TM6SF2 polymorphisms (n = 362)

	CC	CT/TT% / (n) or mean ± SD	*P* value
Age (years)	55.17 ± 12.66	48.88 ± 11.87	0.002*
Men /Women (n)	44.1% (142)/55.9% (180)	52.5% (21)/47.5% (19)	0.313
*Race*			
White	76.7% (247)	75% (30)	0.757
Multiracial (Brown)	13.7% (44)	17.5% (7)	
Black	9.6% (31)	7.5% (3)	
Type 2 diabetes (n)	24.4% (77)	15% (6)	0.186
Dyslipidemia (n)	21.6% (68)	10% (4)	0.086
High-blood pressure (n)	45.3% (143)	27.5% (11)	0.032*
Obesity (BMI ≥ 30) (n)	31.6% (100)	15% (6)	0.030*
BMI	27.7 ± 5.21	26.59 ± 3.24	0.274
Metabolic syndrome (n)	25.3% (80)	7.7% (3)	0.014*
Hypothyroidism (n)	17.4% (55)	17.5% (7)	0.988
Previous alcohol use (n)	8.2% (26)	10% (4)	0.760
HOMA-IR value ≥ 2.5 (n)	53.1% (171)	50% (20)	0.710
HOMA-IR	3.26 ± 4.31	2.69 ± 3.86	0.414
AST (U/L)	63.11 ± 51.51	60.65 ± 42.02	0.897
ALT (U/L)	68.76 ± 52.21	68.33 ± 44.48	0.760
Alkaline phosphatase (U/L)	84.19 ± 35.34	74.05 ± 27.46	0.041*
GGT (U/L)	104.16 ± 113.22	84.26 ± 83.48	0.276
Creatinine (mg/dL)	0.87 ± 0.33	1.01 ± 1.57	0.091
Total cholesterol (mg/dL)	158.24 ± 35.06	147.33 ± 42.32	0.036*
HDL (mg/dL)	52.27 ± 16.12	49.67 ± 15.88	0.516
LDL (mg/dL)	85.69 ± 30.67	79.31 ± 36.08	0.117
Triglycerides (mg/dL)	103.05 ± 54.03	91.05 ± 37.1	0.205
*Liver histology*			
Fibrosis (n) 0–1	33.9% (109)	50% (20)	
2–4	66.1% (213)	50% (20)	0.044*
Inflammation (n) 0–1	46.7% (122)	43.2% (16)	
2–3	53.3% (139)	56.8% (21)	0.689
Steatosis (n) 0	45.3% (145)	40% (16)	
1–3	54.7% (175)	60% (24)	0.524

Chi-square test, Fisher's exact test, Mann–Whitney, and Kruskal–Wallis test

ALT: alanine aminotransferase; AST: aspartate aminotransferase; BMI: body mass index; HCV: hepatitis C virus; HDL: high-density lipoprotein; HOMA-IR: Homeostatic Model of Assessment; LDL: low-density lipoprotein; GGT: gamma glutamyl transferase; SD: standard deviation

*p value < 0.05

On the univariate logistic regression analyses, older age, diabetes/ insulin resistance, dyslipidemia, arterial hypertension, MetS, higher serum levels of AST, ALT, alkaline phosphatase, GGT, and lower serum levels of total cholesterol and LDL were associated with significant liver fibrosis (≥ stage 2 fibrosis) (Table 5). Interestingly, the TM6SF2 CT/TT genotype was associated with a 1.95-fold increased finding of significant liver fibrosis among patients with HCV (*p* = 0.047, OR 1.953, 95% CI 1.009–3.788) (Table 5). In a stepwise multivariate logistic regression analysis, older age (*p* < 0.0001, OR 1.103, 95% CI 1.073–1.134), lower serum levels of total cholesterol (*p* < 0.0001, OR 1.026, 95% CI 1.016–1.036), higher serum levels of ALT (*p* < 0.0001, OR 1.028, 95%CI 1.017–1.038), and alkaline phosphatase (*p* = 0.004, OR 1.019, 95% CI 1.006–1.032) remained independently associated with significant liver fibrosis (Table 5). The TM6SF2 CT genotype was not associated with liver steatosis (*p* = 0.524, OR 1.243, 95% CI 0.636–2.428), or with moderate to intense liver inflammatory activity (*p* = 0.689, OR 1.152, 95%CI 0.575–2.307) on the univariate logistic regression analyses. The same was true for the presence of the G allele of PNPLA3 (*p* = 0.097, OR 1.422, 95% CI 0.937–2.155, and *p* = 0.564, OR 1.143, 95% CI 0.725–1.803, respectively). On the other hand, the presence of MetS (*p* = 0.003, OR 2.227, 95% CI 1.217–4.077), insulin resistance (*p* = 0.025, OR 1.720, 95% CI 1.070–2.756), and higher GGT levels (*p* = 0.013, OR 1.003, 95% CI 1.000–1.006) were independently associated with liver steatosis. The HCV genotype was not associated with any of the liver histology parameters evaluated. In addition, the combination of TM6SF2 and PNPLA3 was not associated with significant liver fibrosis or steatosis (*p* = 0.079 and *p* = 0.161, respectively) on logistic regression analyses.

**Table 5:** Factors associated with significant liver fibrosis in patients with HCV (n = 365)

	Univariate analysis			Multivariate analysis		
Variable	OR	95% CI	*P* value	OR	95% CI	*P* value
Age (years)	1.075	1.054–1.097	< .0001*	1.103	1.073—1.134	< .0001*
Men /Women (n)	1.158	0.753–1.782	0.5037			
Type 2 diabetes (n)	2.532	1.412–4.545	0.0018*			
Dyslipidemia (n)	2.188	1.195–4.000	0.0112*			
High-blood pressure (n)	2.976	1.859–4.762	< .0001*			
Obesity (BMI ≥ 30) (n)	1.086	0.674**–**1.748	0.7358			
Metabolic syndrome (n)	2.299	1.294–4.082	0.0046*			
Hypothyroidism (n)	1.869	0.999**–**3.497	0.0504			
Previous alcohol use (n)	1.089	0.493**–**2.404	0.8325			
HOMA-IR value ≥ 2.5 (n)	1.773	1.149–2.732	0.0096*			
AST (U/L)	1.025	1.016–1.034	< .0001*			
ALT (U/L)	1.025	1.017–1.033	< .0001*	1.028	1.017–1.038	< .0001*
Alkaline phosphatase (U/L)	1.029	1.018–1.040	< .0001*	1.019	1.006–1.032	0.0043*
GGT (U/L)	1.007	1.003–1.010	< .0001*			
Creatinine (mg/dL)	1.183	0.720**–**1.943	0.5065			
Total cholesterol (mg/dL)	0.988	0.982–0.995	0.0002*	0.975	0.965–0.984	< .0001*
HDL (mg/dL)	0.993	0.980–1.006	0.2949			
LDL (mg/dL)	0.987	0.980–0.994	0.0004*			
Triglycerides (mg/dL)	0.997	0.993–1.001	0.1454			
PNPLA3 C / G allele (n)	1.013	0.659–1.557	0.9524			
TM6SF2 CT + TT / CC (n)	1.953	1.009–3.788	0.0471*			

Logistic regression

ALT: alanine aminotransferase; AST: aspartate aminotransferase; BMI: body mass index; CI: confidence interval; GGT: gamma glutamyl transferase; HCV: hepatitis C virus; HDL: high-density lipoprotein; HOMA-IR: Homeostatic Model of Assessment; LDL: low-density lipoprotein; OR: odds ratio

*p value < 0.05

## Discussion

The present study included the largest sample of Brazilians with chronic hepatitis C assessed for the TM6SF2 and PNPLA3 polymorphisms to date. In addition, it added a healthy group to describe these polymorphisms frequencies in subjects without this viral infection. In this cross-sectional clinical study, we could demonstrate that, in this cohort of patients naïve to antiviral therapy, with all stages of hepatic fibrosis, the PNPLA3 GG subjects had lower BMI than CG/CC individuals, and the presence of MetS was more frequent in CC/CG subjects, without any other clinical, biochemical or histological associations. In contrast, in comparison to the CT/TT genotype, the TM6SF2 CC genotype was associated with older age, higher frequency of arterial hypertension, obesity, MetS, higher serum levels of alkaline phosphatase and total cholesterol. Interestingly, the presence of the T allele of TM6SF2 was associated with a 1.95-fold increased finding of significant liver fibrosis in these patients. However, none of the polymorphisms were independently associated with hepatic steatosis or fibrosis.

The TM6SF2 genotypes distribution among patients with HCV was 89% of CC and 11% of CT/TT; results comparable with previously reported data from Milano et al. (2015), whose analysis of 815 subjects with HCV revealed 91.5% for CC and 8.5% for CT/TT [18]. The PNPLA3 CC, CG, and GG genotype frequencies in chronic hepatitis C patients were 51.4%, 41.2%, and 7.4%, respectively. These frequencies were similar to those in the healthy subjects and to previously published data from hepatitis C patients [33]. The distribution of PNPLA3 genotypes among healthy individuals (49.3% CC, 41% CG, and 9.7% GG) was comparable with data obtained from an anterior large systematic review that included 2,504 individuals of different ethnicities, which found frequencies of 56.9%, 33.8%, and 9.3%, respectively [10]. A recent Brazilian study in patients with HCV showed an unusual distribution of PNPLA3 genotypes, with 45.9% CC, 21% CG, and 34.4% GG [28]. The high reported frequency of the G allele in this sample and the discrepancy in the findings of the association of this allele with liver fibrosis in relation to our study may result, at least in part, from the population representativeness of the selected sample, or even that its genetic configuration was undergoing evolution for this specific polymorphism.

Experimental and clinical studies in NAFLD show that the TM6SF2 T allele is associated with decreased TM6SF2 gene expression and consequent hepatic accumulation of lipids and triglycerides. The C allele, on the other hand, plays the opposite role, protecting against liver steatosis, as it stimulates VLDL secretion and increases serum cholesterol levels and cardiovascular risk [11, 15, 34, 35]. Our results were in line with these concepts and with previous publications, with the T allele associated with significant liver fibrosis [18, 28, 36–38]. However, in the multivariate logistic regression analysis, older age and elevated ALT levels (as a surrogate marker of liver inflammation) remained drivers of hepatic fibrosis in this cohort [5]. On the other hand, the relationship of the CC genotype with higher serum total cholesterol levels, arterial hypertension, obesity, and MetS in the present study was compatible with the expected effect of the C allele in the stimulation of hepatic secretion of VLDL [12, 13, 36]. However, we could not analyze the association of the polymorphisms on previous cardiovascular events due to the small number of patients with this feature (only 17 subjects).

When analyzing PNPLA3 polymorphisms, we found no association with liver steatosis or fibrosis in our cohort, despite other compelling data in this direction [24, 25, 39–41]. Huang et al. (2017), found no significant association with the degree of liver fibrosis in 1,080 Asians with HCV [27]. Other studies have also found diverging results [26, 42]. Brazilians are an admixed population, and this could justify our results. However, even in Brazil, the PNPLA3 polymorphisms have also been implicated in liver fibrosis in patients with NAFLD [22, 23, 43] and chronic hepatitis C [28, 38]. However, data from these HCV studies reveal an unexpectedly high frequency of the PNPLA3 G allele [28, 38], and in the report of Magri et al. (2020), the PNPLA3 gene was not in Hardy–Weinberg equilibrium, which might preclude firm conclusions [38]. The differences in the finding of an association of PNPLA3 polymorphisms with liver fibrosis in HCV patients between previously published data [28, 38] and the present study with a larger cohort emphasizes the importance of further investigations on the role of these polymorphisms in chronic hepatitis C patients in Brazil.

In the era of direct-acting antiviral agents with high SVR rates, the identification of genetic polymorphisms and other metabolic characteristics of HCV patients might be of utility for risk stratification after treatment, as in previous studies with interferon based therapy [44, 45]. Indeed, it is important to highlight that the influence of TM6SF2 in the development of significant fibrosis and its association with factors of the MetS might occur independently of the presence of HCV, which demands attention even after the eradication of the virus [36, 46].

The present study has some limitations. The moderate sample size of patients with HCV is a weakness. The lack of information about the participants’ genetic ancestry and other factors, such as the distribution of body fat, may affect results. Also, the single-center cross-sectional study design limits the possibility of external validation of the results and hampers causality assumptions. Lastly, the low number of healthy individuals evaluated for the polymorphisms may not reflect the real frequency of the SNPs in the Brazilian general population.

In conclusion, we demonstrate that, in the univariate logistic regression analysis, the TM6SF2 CC genotype was associated with older age, higher frequency of arterial hypertension, obesity, MetS, higher serum levels of alkaline phosphatase and total cholesterol in comparison to the CT/TT genotype, in the largest sample of Brazilian individuals with chronic hepatitis C to date. Also, the presence of the T allele of TM6SF2 was associated with a 1.95-fold increased finding of significant liver fibrosis in this admixed population. Despite this, neither studied polymorphisms were independently associated with liver steatosis or fibrosis. On the other hand, older age, lower serum levels of total cholesterol and higher serum levels of ALT and alkaline phosphatase were independently associated with liver fibrosis. Other factors seem more influential than these specific polymorphisms in isolation. More studies are warranted to clarify the role of PNPLA3 and TM6SF2 polymorphisms in Brazilians with HCV.

## Data Availability

The datasets analyzed during the current study are available from the corresponding author on reasonable request.

## Abbreviations

AST: Aspartate aminotransferase
ALT: Alanine aminotransferase
BMI: Body mass index
CI: Confidence interval
CVE: Cardiovascular event
GGT: Gamma glutamyl transferase
GWAS: Genome wide association studies
HCV: Hepatitis C virus
HDL: High-density lipoproteins
HSCs: Hepatic stellate cells
HOMA-IR: Homeostatic model of assessment
MetS: Metabolic syndrome
NAFLD: Non-alcoholic fatty liver disease
OR: Odds ratios
PCR: Polymerase chain reaction
PIC: Polymorphic information content
PNPLA3: Patatin-like phospholipase domain containing 3
SD: Standard deviation
SNPs: Single nucleotide polymorphisms
TM6SF2: Transmembrane 6 superfamily member 2
VLDL: Very low density lipoprotein
